# Predictive Value of Semi-Quantitative GeneXpert Categories for Microbiological Outcomes in Pulmonary Tuberculosis

**DOI:** 10.3390/biomedicines14051052

**Published:** 2026-05-06

**Authors:** Elena Cojocaru, Ioana-Adelina Stoian, Radu-Adrian Crișan-Dabija, Ruxandra Cojocaru, Adriana Ignat, Cristian Cojocaru

**Affiliations:** 1 Grigore T. Popa University of Medicine and Pharmacy, 700115 Iasi, Romania; elena.cojocaruu@umfiasi.ro (E.C.); radu.dabija@umfiasi.ro (R.-A.C.-D.); mg-rom-34267@students.umfiasi.ro (R.C.); cristian.cojocaru@umfiasi.ro (C.C.); 2 Clinic of Pulmonary Diseases, 700115 Iasi, Romania; adriana.ignat@pneumo-iasi.ro

**Keywords:** pulmonary tuberculosis, GeneXpert, molecular diagnostics, bacillary load, smear positivity, infectiousness

## Abstract

**Background**: Molecular testing has improved pulmonary tuberculosis (PTB) diagnosis, but the clinical interpretation of semi-quantitative GeneXpert results—particularly at low bacillary loads—remains uncertain. **Methods**: This retrospective study included 167 patients with positive GeneXpert results evaluated at a tertiary pneumology hospital between January and December 2024. Patients were stratified by culture status. Associations between semi-quantitative GeneXpert categories and smear positivity, culture confirmation, and time to culture positivity were evaluated using logistic regression, ROC analysis, and Cox proportional hazards models. **Results**: Increasing GeneXpert categories were associated in a graded association with microbiological positivity. Compared with “Very Low”, the odds of smear positivity were higher for “Medium” (OR 36.00, 95% CI 6.49–199.65) and “High” results (OR 328.50, 95% CI 43.29–2492.98). The probability of culture confirmation increased stepwise (0.60 for “Very Low”, 0.79 for “Low”, 0.93 for “Medium”, and 0.92 for “High”; AUC 0.70), indicating that bacillary load is only one of several determinants of culture positivity. Prior tuberculosis and underweight status were associated with “Very Low” results, while cavitary disease was associated with higher categories. Higher GeneXpert categories were also associated with shorter time to culture positivity. **Conclusions**: Semi-quantitative GeneXpert categories provide clinically relevant information in PTB. “Medium” and “High” results were usually associated with microbiological positivity, whereas “Very Low” results were less reliable and required cautious interpretation. These categories may support early clinical decision-making while culture results are pending.

## 1. Introduction

Tuberculosis (TB) continues to represent a major global health burden, with an estimated 10.7 million incident cases and 1.23 million deaths reported worldwide in 2024 [1]. The disease most often affects the lungs, and pulmonary tuberculosis (PTB) accounts for the majority of presentations. Nevertheless, *Mycobacterium tuberculosis* (*M. tuberculosis*) may involve a wide range of extrapulmonary sites, including the pleura, lymph nodes, abdomen, genitourinary tract, skin, bones, joints and meninges, collectively referred to as extrapulmonary tuberculosis, occurring in approximately 15–20% of patients [2].

Microbiological confirmation remains essential for the diagnosis of PTB. Although culture on solid or liquid media is considered the reference standard because of its high specificity and ability to enable drug-susceptibility testing, its prolonged turnaround time and reduced sensitivity in paucibacillary disease limit its clinical utility. In line with World Health Organization recommendations, rapid molecular assays are therefore used as initial diagnostic tests in individuals with suspected TB [1]. Over the past decade, nucleic acid amplification tests have improved early detection of *M. tuberculosis*, and the GeneXpert platform has shown higher sensitivity than conventional smear microscopy in routine clinical settings [3].

Beyond confirming *M. tuberculosis*, treatment should be guided by a comprehensive microbiological assessment and knowledge of the pathogen’s drug-susceptibility pattern. Accurate identification of the causative organism in serious infections allows clinicians to select targeted regimens and limits the unnecessary use of broad-spectrum antibiotics. Phenotypic or molecular susceptibility testing characterizes the sensitivity and resistance of the isolate to available agents; once results are available, regimens should be de-escalated to focus on the identified organism, thereby limiting costs, toxicity and selection pressure for resistance [4]. For tuberculosis, timely detection of cases enables early initiation of appropriate therapy and curtails transmission and complications. Drug susceptibility testing results should therefore inform the choice of first- and second-line anti-tuberculous agents.

During the COVID-19 pandemic, there was a surge in inappropriate antibiotic use—including empirical prescribing, self-medication and increased consumption of reserve agents—despite the low prevalence of bacterial co-infection; WHO and observational studies have warned that such practices accelerate the emergence of antimicrobial resistance and multidrug-resistant pathogens [5].

Despite these advances, uncertainty remains in the interpretation of semi-quantitative GeneXpert results. In patients with negative Ziehl–Neelsen smear microscopy and pending culture confirmation, decisions regarding treatment initiation, respiratory isolation and assessment of infectiousness often rely heavily on molecular findings. This issue is particularly relevant in patients with previous PTB or residual cavitary lesions, findings that are common in regions with historically high TB incidence and in which molecular positivity does not always indicate active disease but may instead reflect nonviable bacillary DNA [6].

In this context, GeneXpert categories reported as “Very Low” or “Low” remain difficult to interpret with respect to true microbiological positivity and transmission risk [7]. Evidence on the clinical meaning of these semi-quantitative categories remains limited, and their implications for routine patient management are not fully defined.

However, integrated analyses that combine semi-quantitative GeneXpert categories with both cross-sectional microbiological outcomes and time to culture positivity remain limited, particularly in routine-care European settings where pragmatic clinical interpretation is particularly important.

This study evaluated the predictive value of semi-quantitative GeneXpert categories for culture positivity and smear status in patients with suspected PTB, with a focus on their clinical relevance within a molecularly confirmed cohort.

## 2. Materials and Methods

### 2.1. Study Design and Population

We conducted a retrospective observational study at the Molecular Biology Laboratory of the Clinic of Pulmonary Diseases, Iași, Romania, a tertiary referral centre for TB. We reviewed the medical records of patients assessed for suspected PTB between 1 January and 31 December 2024. A total of 167 adult patients with a positive GeneXpert result for *M. tuberculosis* complex met the inclusion criteria, and baseline culture data were available for all patients.

Demographic variables (age and sex) were recorded for all participants. Only patients with a positive GeneXpert MTB/RIF Ultra result were included. The analysis was therefore restricted to a molecularly confirmed cohort, thereby allowing evaluation of the clinical and microbiological correlates of semi-quantitative GeneXpert categories. As a result, the study was not designed to assess diagnostic performance, and measures such as sensitivity and specificity were not evaluated. Smoking status was recorded as part of the baseline clinical evaluation. Radiological findings were obtained from imaging reports and included disease distribution (unilateral versus bilateral involvement) and the presence of cavitary lesions. All patients underwent HIV testing and were HIV-negative. The exclusion criteria were as follows: (i) individuals younger than 18 years, (ii) cases with extrapulmonary specimens, (iii) patients already receiving anti-tuberculosis therapy at the time of sampling, and (iv) records with incomplete or missing culture data. These exclusions were applied to ensure a consistent, well-defined cohort suitable for retrospective analysis.

### 2.2. Sample Size Calculation and Power of the Study

Following recommendations for cross-sectional studies [8], we calculated the minimum sample size required for our retrospective cohort. Using the CDC Epi-Info software (Version 7.2) for population surveys with a 95% confidence level, a 5% margin of error and an expected frequency of 50%, at least 384 individuals would be needed to ensure adequate statistical power.

In our study group, the estimated margin of error was approximately 7.6%. Although smaller than the recommended size for a general population survey, the number of cases and culture-positive events in our cohort was sufficient to detect clinically meaningful associations. Power calculations for logistic regression indicate that with 167 participants (142 culture-positive events), the study has >80% power (α = 0.05) to detect odds ratios ≥3 for culture positivity between GeneXpert categories. We acknowledge that the reduced sample leads to wider confidence intervals and address this limitation in the Discussion section.

### 2.3. Microbiological Procedures

Sputum specimens were processed according to routine laboratory procedures. Molecular detection of *M. tuberculosis* complex was performed using the GeneXpert IV platform (Cepheid, Sunnyvale, CA, USA) with the GeneXpert MTB/RIF (*M. tuberculosis*/Rifampicin) Ultra assay, in accordance with the manufacturer’s instructions. The fully automated GeneXpert^®^ Dx 6.4 Software controls sample processing, DNA extraction, amplification and real-time detection and generates semi-quantitative bacillary load categories (“High”, “Medium”, “Low” or “Very Low”) together with rifampicin resistance results. All specimens were further examined by Ziehl–Neelsen smear microscopy for acid-fast bacilli and cultured on Löwenstein–Jensen solid medium and in the BACTEC Mycobacteria Growth Indicator Tube (MGIT 960) system containing modified Middlebrook 7H9 broth (liquid culture), following standard laboratory procedures. Culture results were considered the reference standard for microbiological confirmation of *M. tuberculosis*.

### 2.4. Study Groups and Outcomes

Patients with positive GeneXpert results were stratified according to final culture outcome into culture-positive and culture-negative groups. The primary outcomes were culture positivity for *M. tuberculosis*, smear microscopy status and time to culture positivity. Associations between semi-quantitative GeneXpert categories and microbiological outcomes were further explored.

### 2.5. Statistical Analysis

Continuous variables are reported as mean ± standard deviation or median and were compared using Student’s *t*-test or the Mann–Whitney U test, as appropriate. Categorical variables are presented as counts (%) and were compared using the χ^2^ test or Fisher’s exact test, with Fisher’s exact test used when expected cell counts were <5. A two-sided *p*-value < 0.05 was considered statistically significant.

The association between semi-quantitative GeneXpert categories and smear positivity was assessed using univariable logistic regression, with the “Very Low” category as the reference group; results are presented as odds ratios (ORs) with 95% confidence intervals (CI). Logistic regression was also used to estimate the probability of baseline culture positivity and to assess discrimination using the area under the receiver operating characteristic curve (AUC). Smear positivity was summarized descriptively for each semi-quantitative GeneXpert category; proportions were calculated as the number of smear-positive patients divided by the total number of patients in each category.

Time to culture positivity was analyzed using Kaplan–Meier methods with log-rank testing and Cox proportional hazards models to estimate hazard ratios. The proportional hazards assumption was evaluated using Schoenfeld residuals and graphical inspection. Additional univariable logistic models explored factors associated with a “Very Low” result. Correlation between GeneXpert category and culture positivity was assessed using Spearman’s and Kendall’s coefficients. Analyses involving culture status were performed on available cases. All analyses were conducted using R statistical software (version 4.3.2; R Foundation for Statistical Computing, Vienna, Austria).

### 2.6. Ethical Considerations

The investigation was conducted in accordance with the principles of the Declaration of Helsinki and was approved by the Ethics Committee of the Clinic of Pulmonary Diseases, Iasi, Romania (Approval No. 134/26 January 2026). The requirement for informed consent was waived by the Ethics Committee due to the retrospective design of the study and the use of anonymized data.

## 3. Results

A total of 167 patients with positive GeneXpert results were included, and baseline culture data were available for all patients. The mean age of the study population was 47.9 ± 15.3 years, and 79.8% were male. Based on final bacteriological results, 142 patients (85.0%) were culture-positive, whereas 25 (15.0%) were culture-negative.

Baseline demographic, clinical and radiological characteristics stratified by culture outcome are detailed in Table 1.

The two groups were comparable in terms of age and sex distribution, indicating no substantial demographic imbalance. Culture-positive patients were more likely to be classified into higher GeneXpert semi-quantitative categories (“Medium” and “High”), whereas the majority of culture-negative cases were classified as “Low” or “Very Low”. Cavitary pulmonary lesions and underweight status showed no significant differences between groups (Table 1). Smoking status and radiological extent (unilateral versus bilateral disease) were explored as potential confounders. No statistically significant differences were observed between culture-positive and culture-negative groups (*p* = 0.156 and *p* = 0.202, respectively). The distribution of semi-quantitative GeneXpert categories differed between culture-positive and culture-negative patients, with negative cultures occurring most frequently in the “Very Low” group and culture positivity increasing across the higher categories. A modest but statistically significant positive correlation between GeneXpert category and baseline culture positivity was observed (Spearman ρ = 0.26, *p* < 0.001; Kendall τ = 0.24, *p* < 0.001).

A clear positive association was observed between increasing GeneXpert signal and smear positivity. In a logistic regression model using the “Very Low” category as the reference, the odds of smear positivity rose across the “Low”, “Medium” and “High” categories (Table 2). The “Low” category showed a modest, non-significant increase in smear positivity compared with “Very Low”, whereas both the “Medium” and “High” categories were associated with much higher ORs reaching statistical significance.

**Figure 1:**
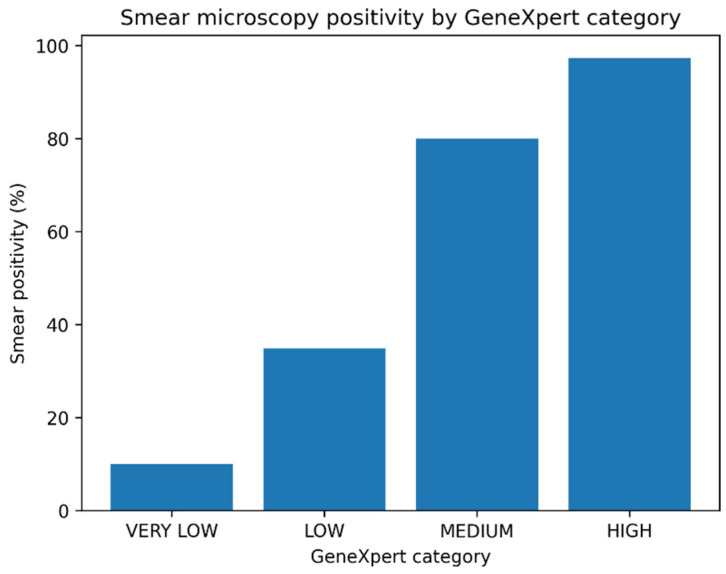
Smear microscopy positivity by GeneXpert category. Bars represent the proportion of smear-positive patients within each semi-quantitative GeneXpert category, calculated as the number of smear-positive cases divided by the total number of patients in each category. The association between categories and smear positivity was assessed using a univariable logistic regression model (see Methods and Table 2).

Figure 1 shows that smear positivity was concentrated in the “Medium” and “High” categories, whereas most “Very Low” and “Low” results were smear-negative.

**Figure 2:**
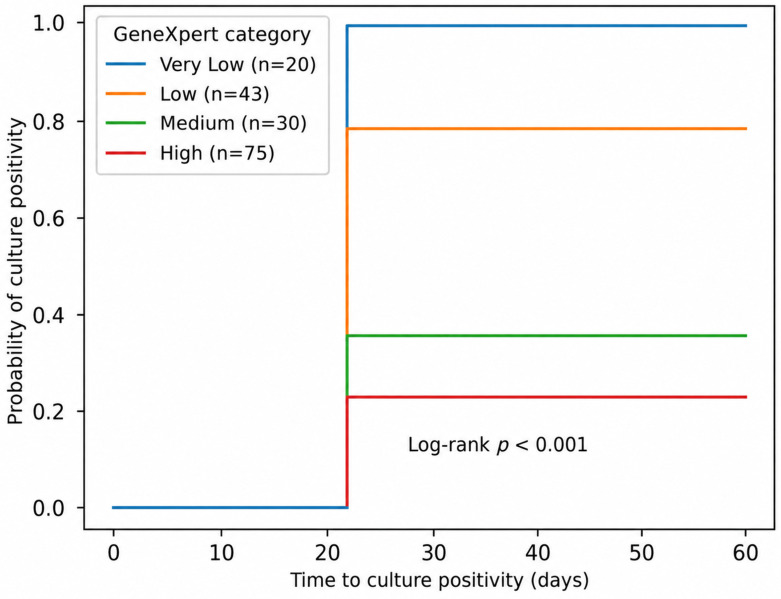
Time to culture positivity across semi-quantitative GeneXpert categories.

Time to culture positivity also differed significantly across the GeneXpert semi-quantitative categories (Figure 2). Kaplan–Meier analysis showed early separation between the curves, with the “Medium” and “High” groups showing earlier culture positivity than the “Very Low” and “Low” groups. By day 21, the cumulative probability of culture positivity remained higher in the “Medium” and “High” GeneXpert categories, in keeping with the overall pattern of earlier culture detection in the “Medium” and “High” groups. The time to culture positivity was shortest in the “High” category, and these differences were statistically significant (log-rank *p* < 0.001). The apparent rise in culture positivity after day 21 reflects the distribution of time to positivity: several cultures turned positive between days 14 and 21, resulting in a stepwise increase in the cumulative percentage. This pattern is consistent with the growth kinetics of *M. tuberculosis* and does not reflect any change in methods or sampling.

A Cox proportional hazards model was used to evaluate the association between GeneXpert semi-quantitative categories and time to culture positivity, with the “Very Low” group as the reference (Table 3). There was no significant difference between the “Low” and “Very Low” groups. In contrast, hazard ratios for the “Medium” and “High” categories were substantially above one, indicating a progressively faster rate of culture positivity with increasing GeneXpert values. Overall, higher semi-quantitative categories were associated with earlier culture detection.

To explore whether prior TB, cavitary disease and nutritional status influence GeneXpert results, we performed univariable logistic regression analyses. A history of PTB and being underweight were associated with increased odds of a ‘Very Low’ GeneXpert result, whereas the presence of cavitary lesions was associated with decreased odds of a ‘Very Low’ result. These associations and their statistical significance are summarized in Table 4.

**Figure 3:**
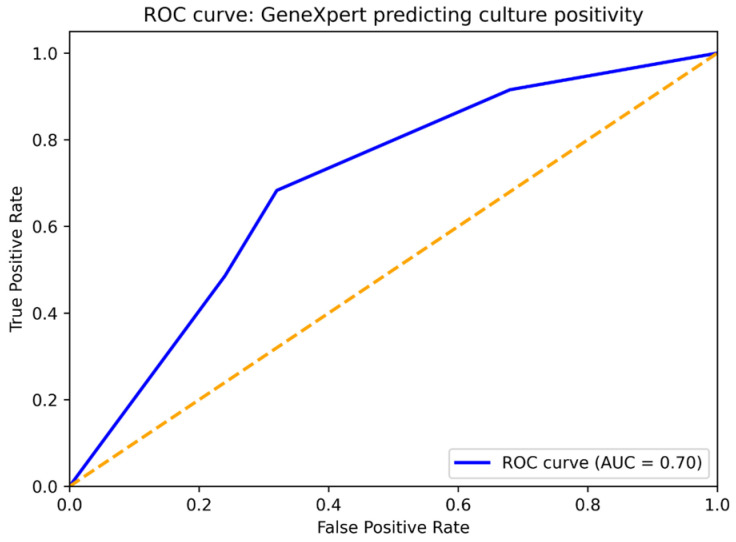
ROC curve for the prediction of culture positivity by GeneXpert category. ROC = Receiver Operating Characteristic; AUC = Area Under the Curve. The dashed diagonal line represents the line of no discrimination (AUC = 0.5).

The receiver operating characteristic (ROC) curve in Figure 3 summarizes the ability of the semi-quantitative GeneXpert assay to predict baseline culture positivity. The overall discrimination was moderate (AUC 0.70). “Medium” and “High” results were strongly associated with culture positivity and demonstrated high sensitivity, whereas “Low” and “Very Low” results were less predictive and included cases in which mycobacterial DNA was detected despite a negative culture. These findings suggest that when the GeneXpert shows a high semi-quantitative category, it most often corresponds to culture-confirmed disease. Conversely, a “Very Low” result is more likely to reflect a very low bacterial burden or DNA from dead organisms, rather than active, culture-positive disease.

The probability of culture positivity increased stepwise across the GeneXpert categories, from “Very Low” to “High”. Model-based estimates were similar to the observed data (Table 5).

**Figure 4:**
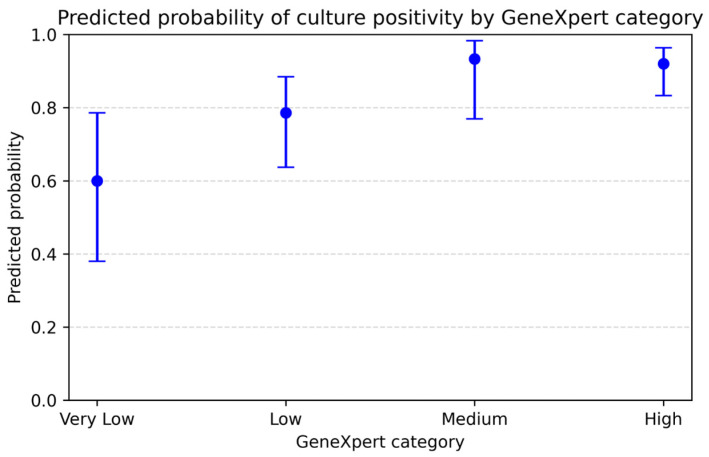
Predicted probability of culture positivity by GeneXpert category.

Figure 4 illustrates the same trend across categories.

## 4. Discussion

In our retrospective cohort of GeneXpert-positive PTB patients, we demonstrated a consistent and clinically relevant relationship between the semi-quantitative GeneXpert categories and conventional bacteriological results. Across all analytical approaches used, GeneXpert categories showed a stepwise association with smear positivity, culture confirmation, and time to culture positivity. Overall, the semi-quantitative GeneXpert categories provided a reliable proxy for bacillary load, although some subgroups were small, particularly the “Very Low” category. The consistency across analyses supports the robustness of the findings.

In our cohort, microbiological positivity increased across the GeneXpert categories. The wide CIs in the highest categories are most likely related to the small number of patients in these strata and the marked gradient observed in the data, rather than model instability. “Medium” and “High” results were more frequent among smear-positive and culture-positive cases, whereas “Very Low” and “Low” results were predominantly smear-negative and showed substantially lower culture yield. Time-to-event analysis further reinforced this pattern, demonstrating earlier culture positivity in the “Medium” and “High” strata and progressively delayed positivity in the “Very Low” and “Low” groups.

Our findings are consistent with accumulating evidence that semi-quantitative outputs from the Xpert platform reflect underlying bacillary burden. In a recent multicentre analysis, Méchaï et al. demonstrated a strong association between GeneXpert cycle-threshold values, smear grading and time to culture detection [9]. Fradejas et al. reported lower cycle threshold (Ct) values in patients with higher smear grades and shorter time to culture positivity [10]. Similar observations have been reported for Xpert Ultra, where the higher semi-quantitative categories tend to be culture-positive more often and are regarded as more likely to reflect infectious disease [11]. A similar pattern was observed in our cohort.

Our results also highlight several aspects that are less frequently addressed in previous studies. The “Very Low” category, in particular, showed a different pattern in our cohort and included a substantial number of culture-negative cases. These findings suggest that low-level molecular positivity may not necessarily indicate active disease, even when culture is negative, and should therefore be interpreted in conjunction with clinical findings.

In the ROC analysis, discriminatory performance was moderate (AUC 0.70) and should be interpreted with caution, as this level of discrimination limits its use as a standalone decision tool and may lead to misclassification in individual cases. These findings are particularly relevant when interpreting low-level positive GeneXpert results. In smear-negative cases, disagreement between GeneXpert and conventional bacteriology is not unexpected and may reflect the limited sensitivity of microscopy, low bacillary viability or pre-analytical factors affecting culture recovery. Nucleic acid amplification tests detect mycobacterial DNA regardless of bacterial viability. Persistent Xpert positivity after treatment has been described and is generally attributed to the detection of non-viable bacillary DNA [12].

In our cohort, semi-quantitative GeneXpert categories were closely related to microbiological confirmation. ‘Medium’ and ‘High’ results were usually culture-positive and may help inform early treatment decisions. ‘Low’ results should be interpreted in the clinical context. ‘Very Low’ results were often culture-negative and should be interpreted with caution, particularly in patients with a history of tuberculosis or underlying lung disease, as further microbiological evaluation or follow-up may be needed. These findings are consistent with previous reports showing that higher semi-quantitative categories are more often associated with microbiological confirmation [13,14].

From a clinical and infection-control perspective, these observations have practical implications. Patients with “Medium” or “High” GeneXpert results are likely to harbor a higher bacillary burden and to remain culture-positive for longer periods, which may have implications for treatment urgency and infection-control measures [9,15]. Conversely, patients with “Low” or “Very Low” results, particularly those with previous TB, may require careful diagnostic confirmation before clinical decision-making, with short-interval microbiological follow-up prior to definitive therapeutic decisions, provided that the clinical condition permits. Incorporation of semi-quantitative GeneXpert categories into triage algorithms may therefore improve early risk stratification in routine practice [16,17]. Our results further support the role of semi-quantitative GeneXpert reporting in early risk stratification and initial management of suspected PTB.

Based on these findings, a simple practical approach may be considered in routine care. “Medium” and “High” GeneXpert results could support early clinical decisions, including prompt treatment initiation and infection-control measures, given their strong association with microbiological confirmation. In contrast, “Low” and especially “Very Low” results should be interpreted more cautiously and correlated with clinical, radiological and follow-up microbiological data before definitive decisions are made.

An additional conceptual insight from this work is the biological divergence between molecular detection and bacillary viability at the lower end of the positivity spectrum. While higher GeneXpert categories closely tracked culture behavior, the “Very Low” category showed substantial heterogeneity. This observation highlights a recognized limitation of molecular assays and highlights the importance of integrated diagnostic interpretation rather than reliance on a single modality.

These findings highlight the broader need for careful antibiotic stewardship. After cultures and susceptibility results are available, treatment should be narrowed to the most appropriate drug, thereby sparing patients unnecessary broad-spectrum agents and reducing the selection pressure that drives resistance [18].

Transmission outcomes were not directly evaluated in this study and should be addressed in future prospective investigations.

This study has several strengths compared with previous reports. We integrated semi-quantitative GeneXpert categories with multiple microbiological endpoints, including smear status, culture confirmation and time to culture positivity, providing a more comprehensive evaluation. In addition, survival analysis made it possible to explore how GeneXpert categories relate to the timing of culture positivity. The fact that the study was carried out in a tertiary care setting supports the clinical relevance of the findings.

This retrospective, single-center study, including only GeneXpert-positive patients may limit generalizability and introduce potential selection bias. Some subgroup analyses were based on small numbers, resulting in wide CIs. Given the relatively small sample size and the limited number of events in some subgroups, multivariable models were not performed to avoid overfitting and unstable estimates; consequently, residual confounding cannot be excluded. Although all eligible patients were included, the study population (N = 167) was smaller than the 384 participants recommended to achieve a 5% margin of error. Consequently, the margin of error is approximately 7.6%, and the study may be underpowered to detect small effect sizes. However, power calculations indicate that the number of culture-positive events provides >80% power to detect odds ratios ≥3 for the associations examined.

In our cohort, the semi-quantitative GeneXpert categories were consistent with the microbiological findings. “Medium” and “High” results were usually culture-positive and tended to turn positive earlier, while “Very Low” results were more often culture-negative and more difficult to interpret. This was especially the case in patients with previous TB or undernutrition. In routine clinical practice, these results may be useful for early assessment while waiting for culture confirmation. Further prospective studies, including Ct values and transmission data, would help clarify how these results should be used in routine care.

## 5. Conclusions

Our study demonstrates that semi-quantitative GeneXpert categories correlate strongly with smear microscopy, culture positivity, and time to culture detection. ‘Medium’ and ‘High’ categories were consistently associated with culture confirmation and earlier growth, supporting their use as a proxy for higher bacillary burden and probable active disease. In contrast, ‘Low’ categories required integration with clinical and radiological findings, and ‘Very Low’ categories often corresponded to culture-negative patients, underscoring the need for cautious interpretation in such cases.

By linking the semi-quantitative GeneXpert output to well-established microbiological markers, our findings suggest that these categories may help inform early treatment initiation, infection-control measures and patient counseling while awaiting culture results. This correlation underscores the value of rapid molecular assays not only for diagnosis but also for stratifying the risk of infectiousness and guiding antimicrobial therapy. Appropriate selection of antibiotics and de-escalation based on culture and susceptibility results remain essential to minimize the emergence of antimicrobial resistance. Further prospective studies integrating cycle-threshold values and transmission outcomes are warranted to refine the clinical utility of semi-quantitative GeneXpert categories.

## Figures and Tables

**Table 1:** Baseline characteristics according to culture status.

Characteristic	Culture-Positive (*n* = 142)	Culture-Negative (*n* = 25)	*p*-Value
Age, years	48.0 ± 15.4	48.0 ± 15.0	0.990
Male sex, n (%)	114 (80.3)	19 (76.0)	0.624
Smoking status, n (%)	92 (65.2)	12 (48.0)	0.156
Bilateral pulmonary involvement, n (%)	111 (78.2)	16 (64.0)	0.202
GeneXpert semi-quantitative result, n (%) “Very Low” “Low” “Medium” “High”			0.001 †
	12 (8.5)	8 (32.0)	
	33 (23.2) 28 (19.7)	9 (36.0) 2 (8.0)	
	69 (48.6)	6 (24.0)	
Smear microscopy, n (%) Positive			
	107 (75.4)	6 (24.0)	<0.0001
Prior TB history, n (%)	24 (16.9)	7 (28.0)	0.261
Pulmonary cavitation, n (%)	83 (58.5)	12 (48.0)	0.331
Underweight, n (%)	64 (45.1)	12 (48.0)	0.786

Legend: Continuous variables were compared using Welch’s *t*-test. Categorical variables were compared using the χ^2^ test or Fisher’s exact test when expected cell counts were <5. n, number of clinical samples; †, global comparison across GeneXpert categories using the χ^2^ test; TB, tuberculosis. Percentages were calculated on available data.

**Table 2:** Univariable logistic regression for smear microscopy positivity according to semi-quantitative GeneXpert category (reference: “Very Low”).

GeneXpert Category	OR	95% CI	*p*-Value
“Low”	4.50	0.91–22.19	0.065
“Medium”	36.00	6.49–199.65	<0.001
“High”	328.50	43.29–2492.98	<0.001

Legend: ORs, odds ratios; CI, confidence interval.

**Table 3:** Cox proportional hazards model for time to culture positivity, with “Very Low” as reference.

Predictor (“Very Low” as Reference)	HR	95% CI	*p*-Value
“Low”	5.86	0.76–45.46	0.090
“Medium”	12.42	1.67–92.19	0.014
“High”	13.81	1.90–100.34	0.009

Legend: HR, hazard ratios; CI, confidence interval.

**Table 4:** Univariable predictors of a “Very Low” GeneXpert result.

Predictor (Yes vs. No)	ORs (95% CI)	*p*-Value
Prior TB diagnosis	2.31 (1.04–5.14)	0.039
Cavitary lesion	0.28 (0.09–0.86)	0.026
Underweight	1.94 (1.01–3.73)	0.047

Legend: ORs, odds ratios; CI, confidence interval; TB, tuberculosis.

**Table 5:** Probability of baseline culture positivity by GeneXpert semi-quantitative category.

GeneXpert Category	n	Culture- Positive (n)	Observed Probability	95% CI (Wilson)	Estimated Probability (Logistic)	95% CI (Model)
“Very Low”	20	12	0.600	0.387–0.781	0.600	0.380–0.786
“Low”	42	33	0.786	0.641–0.883	0.786	0.637–0.885
“Medium”	30	28	0.933	0.787–0.982	0.933	0.769–0.983
“High”	75	69	0.920	0.836–0.963	0.920	0.833–0.964

Legend: n, number of clinical samples; CI, confidence interval.

## Data Availability

The original contributions presented in this study are included in the article. Further inquiries can be directed to the corresponding author.

## Abbreviations

The following abbreviations are used in this manuscript:

AUC	Area Under the Receiver Operating Characteristic Curve
CI	Confidence Interval
Ct	Cycle Threshold
DNA	Deoxyribonucleic Acid
DX	Diagnostic
HR	Hazard Ratio
*M. tuberculosis*	*Mycobacterium tuberculosis*
MTB/RIF	*Mycobacterium tuberculosis*/Rifampicin
OR/ORs	Odds Ratio/Odds Ratios
PTB	Pulmonary Tuberculosis
R	R Statistical Computing Environment
ROC	Receiver Operating Characteristic
TB	Tuberculosis
WHO	World Health Organization
